# Supplementary material for: Green Fabrication of Silver Nanoparticles, Statistical Process Optimization, Characterization, and Molecular Docking Analysis of Their Antimicrobial Activities onto Cotton Fabrics
Source: J Funct Biomater. 2024 Nov 21;15(12):354. doi: 10.3390/jfb15120354 (PMC11728425; doi:10.3390/jfb15120354)
Supplement: Supplementary file 1 [file jfb-15-00354-s001.zip › jfb-3285343-supplementary.pdf]

Supplementary Table S1. Pharmacokinetics properties of predicted compound resulted from AgNPs loaded on cotton fabrics

Pharmacokinetics and Drug likeness properties	
GI absorption	Low
BBB permeant	No
P-gp substrate	No
CYP1A2 inhibitor	No
CYP2C19 inhibitor	No
CYP2C9 inhibitor	No
CYP2D6 inhibitor	No
CYP3A4 inhibitor	No
Log K_p (skin permeation)	-9.49 cm/s
Lipinski	Yes;
Veber	Yes
Egan	Yes
Bioavailability Score	0.55
